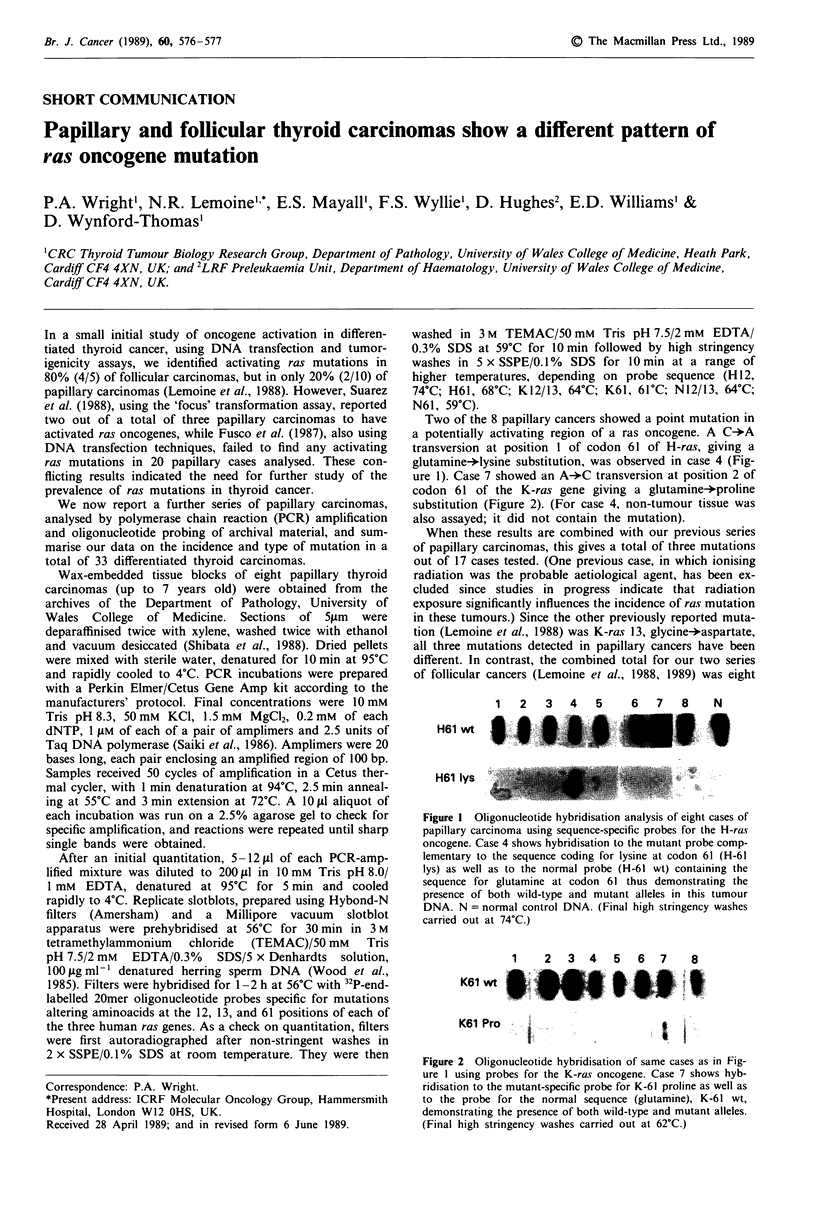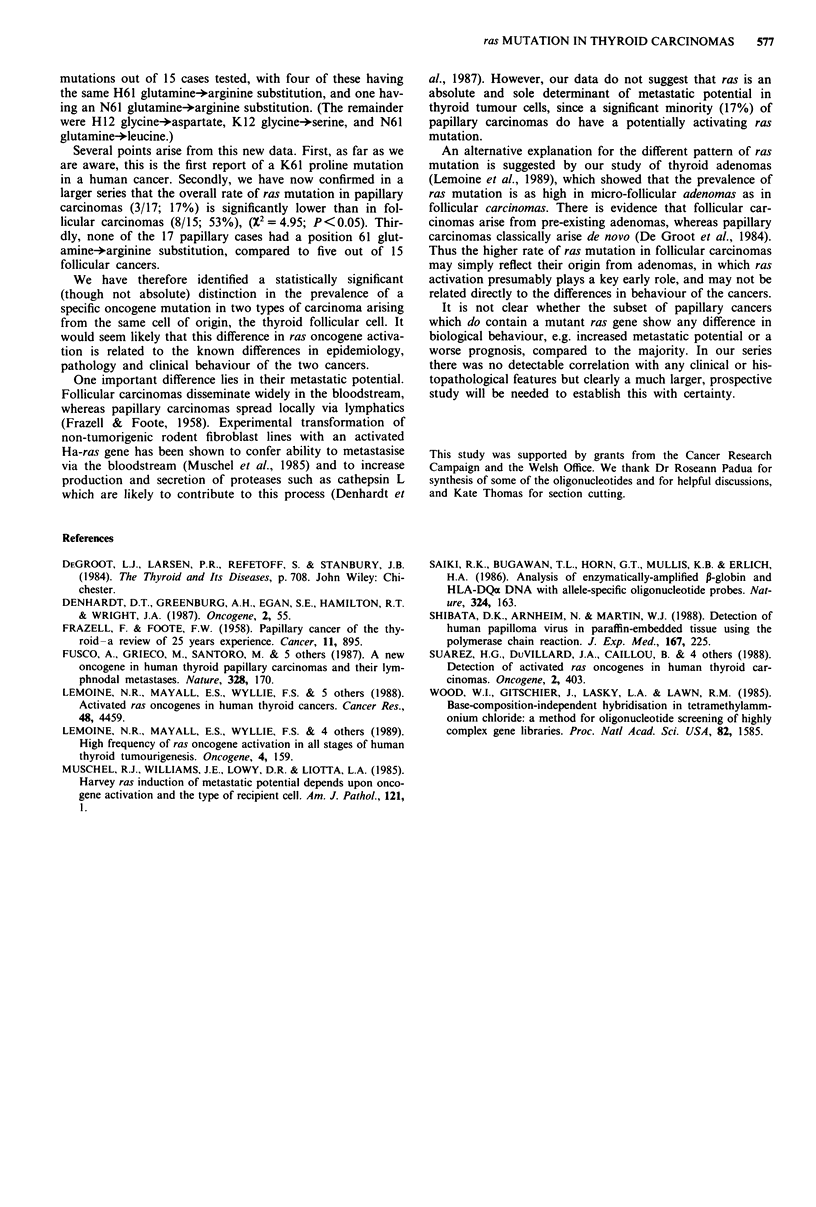# Papillary and follicular thyroid carcinomas show a different pattern of ras oncogene mutation.

**DOI:** 10.1038/bjc.1989.316

**Published:** 1989-10

**Authors:** P. A. Wright, N. R. Lemoine, E. S. Mayall, F. S. Wyllie, D. Hughes, E. D. Williams, D. Wynford-Thomas

**Affiliations:** CRC Thyroid Tumour Biology Research Group, Department of Pathology, University of Wales College of Medicine, Heath Park, Cardiff, UK.

## Abstract

**Images:**


*Br. J. Cancer* (1989), **60**, 576–577
© The Macmillan Press Ltd., 1989

### SHORT COMMUNICATION

### Papillary and follicular thyroid carcinomas show a different pattern of *ras* oncogene mutation

P.A. Wright[1], N.R. Lemoine[1,*], E.S. Mayall[1], F.S. Wyllie[1], D. Hughes[2], E.D. Williams[1] & D. Wynford-Thomas[1]

[1]*CRC Thyroid Tumour Biology Research Group, Department of Pathology, University of Wales College of Medicine, Heath Park, Cardiff CF4 4XN, UK; and* [2]*LRF Preleukaemia Unit, Department of Haematology, University of Wales College of Medicine, Cardiff CF4 4XN, UK.*

In a small initial study of oncogene activation in differentiated thyroid cancer, using DNA transfection and tumorigenicity assays, we identified activating *ras* mutations in 80% (4/5) of follicular carcinomas, but in only 20% (2/10) of papillary carcinomas (Lemoine *et al*., 1988). However, Suarez *et al*. (1988), using the 'focus' transformation assay, reported two out of a total of three papillary carcinomas to have activated *ras* oncogenes, while Fusco *et al*. (1987), also using DNA transfection techniques, failed to find any activating *ras* mutations in 20 papillary cases analysed. These conflicting results indicated the need for further study of the prevalence of *ras* mutations in thyroid cancer.

We now report a further series of papillary carcinomas, analysed by polymerase chain reaction (PCR) amplification and oligonucleotide probing of archival material, and summarise our data on the incidence and type of mutation in a total of 33 differentiated thyroid carcinomas.

Wax-embedded tissue blocks of eight papillary thyroid carcinomas (up to 7 years old) were obtained from the archives of the Department of Pathology, University of Wales College of Medicine. Sections of 5μm were deparaffinised twice with xylene, washed twice with ethanol and vacuum desiccated (Shibata *et al*., 1988). Dried pellets were mixed with sterile water, denatured for 10 min at 95°C and rapidly cooled to 4°C. PCR incubations were prepared with a Perkin Elmer/Cetus Gene Amp kit according to the manufacturers' protocol. Final concentrations were 10 mM Tris pH 8.3, 50 mM KCl, 1.5 mM $MgCl_2$, 0.2 mM of each dNTP, 1 μM of each of a pair of amplimers and 2.5 units of Taq DNA polymerase (Saiki *et al*., 1986). Amplimers were 20 bases long, each pair enclosing an amplified region of 100 bp. Samples received 50 cycles of amplification in a Cetus thermal cycler, with 1 min denaturation at 94°C, 2.5 min annealing at 55°C and 3 min extension at 72°C. A 10 μl aliquot of each incubation was run on a 2.5% agarose gel to check for specific amplification, and reactions were repeated until sharp single bands were obtained.

After an initial quantitation, 5–12 μl of each PCR-amplified mixture was diluted to 200 μl in 10 mM Tris pH 8.0/1 mM EDTA, denatured at 95°C for 5 min and cooled rapidly to 4°C. Replicate slotblots, prepared using Hybond-N filters (Amersham) and a Millipore vacuum slotblot apparatus were prehybridised at 56°C for 30 min in 3 M tetramethylammonium chloride (TEMAC)/50 mM Tris pH 7.5/2 mM EDTA/0.3% SDS/5 × Denhardts solution, 100 μg $ml^{-1}$ denatured herring sperm DNA (Wood *et al*., 1985). Filters were hybridised for 1–2 h at 56°C with $^{32}P$-end-labelled 20mer oligonucleotide probes specific for mutations altering aminoacids at the 12, 13, and 61 positions of each of the three human *ras* genes. As a check on quantitation, filters were first autoradiographed after non-stringent washes in 2 × SSPE/0.1% SDS at room temperature. They were then washed in 3 M TEMAC/50 mM Tris pH 7.5/2 mM EDTA/0.3% SDS at 59°C for 10 min followed by high stringency washes in 5 × SSPE/0.1% SDS for 10 min at a range of higher temperatures, depending on probe sequence (H12, 74°C; H61, 68°C; K12/13, 64°C; K61, 61°C; N12/13, 64°C; N61, 59°C).

Two of the 8 papillary cancers showed a point mutation in a potentially activating region of a ras oncogene. A C→A transversion at position 1 of codon 61 of H-*ras*, giving a glutamine→lysine substitution, was observed in case 4 (Figure 1). Case 7 showed an A→C transversion at position 2 of codon 61 of the K-*ras* gene giving a glutamine→proline substitution (Figure 2). (For case 4, non-tumour tissue was also assayed; it did not contain the mutation).

When these results are combined with our previous series of papillary carcinomas, this gives a total of three mutations out of 17 cases tested. (One previous case, in which ionising radiation was the probable aetiological agent, has been excluded since studies in progress indicate that radiation exposure significantly influences the incidence of *ras* mutation in these tumours.) Since the other previously reported mutation (Lemoine *et al*., 1988) was K-*ras* 13, glycine→aspartate, all three mutations detected in papillary cancers have been different. In contrast, the combined total for our two series of follicular cancers (Lemoine *et al*., 1988, 1989) was eight

**Figure 1** Oligonucleotide hybridisation analysis of eight cases of papillary carcinoma using sequence-specific probes for the H-*ras* oncogene. Case 4 shows hybridisation to the mutant probe complementary to the sequence coding for lysine at codon 61 (H-61 lys) as well as to the normal probe (H-61 wt) containing the sequence for glutamine at codon 61 thus demonstrating the presence of both wild-type and mutant alleles in this tumour DNA. N = normal control DNA. (Final high stringency washes carried out at 74°C.)

**Figure 2** Oligonucleotide hybridisation of same cases as in Figure 1 using probes for the K-*ras* oncogene. Case 7 shows hybridisation to the mutant-specific probe for K-61 proline as well as to the probe for the normal sequence (glutamine), K-61 wt, demonstrating the presence of both wild-type and mutant alleles. (Final high stringency washes carried out at 62°C.)

Correspondence: P.A. Wright.
*Present address: ICRF Molecular Oncology Group, Hammersmith Hospital, London W12 0HS, UK.
Received 28 April 1989; and in revised form 6 June 1989.

mutations out of 15 cases tested, with four of these having the same H61 glutamine→arginine substitution, and one having an N61 glutamine→arginine substitution. (The remainder were H12 glycine→aspartate, K12 glycine→serine, and N61 glutamine→leucine.)

Several points arise from this new data. First, as far as we are aware, this is the first report of a K61 proline mutation in a human cancer. Secondly, we have now confirmed in a larger series that the overall rate of *ras* mutation in papillary carcinomas (3/17; 17%) is significantly lower than in follicular carcinomas (8/15; 53%), ($\chi^2 = 4.95$; $P < 0.05$). Thirdly, none of the 17 papillary cases had a position 61 glutamine→arginine substitution, compared to five out of 15 follicular cancers.

We have therefore identified a statistically significant (though not absolute) distinction in the prevalence of a specific oncogene mutation in two types of carcinoma arising from the same cell of origin, the thyroid follicular cell. It would seem likely that this difference in *ras* oncogene activation is related to the known differences in epidemiology, pathology and clinical behaviour of the two cancers.

One important difference lies in their metastatic potential. Follicular carcinomas disseminate widely in the bloodstream, whereas papillary carcinomas spread locally via lymphatics (Frazell & Foote, 1958). Experimental transformation of non-tumorigenic rodent fibroblast lines with an activated Ha-*ras* gene has been shown to confer ability to metastasise via the bloodstream (Muschel *et al.*, 1985) and to increase production and secretion of proteases such as cathepsin L which are likely to contribute to this process (Denhardt *et al.*, 1987). However, our data do not suggest that *ras* is an absolute and sole determinant of metastatic potential in thyroid tumour cells, since a significant minority (17%) of papillary carcinomas do have a potentially activating *ras* mutation.

An alternative explanation for the different pattern of *ras* mutation is suggested by our study of thyroid adenomas (Lemoine *et al.*, 1989), which showed that the prevalence of *ras* mutation is as high in micro-follicular *adenomas* as in follicular *carcinomas*. There is evidence that follicular carcinomas arise from pre-existing adenomas, whereas papillary carcinomas classically arise *de novo* (De Groot *et al.*, 1984). Thus the higher rate of *ras* mutation in follicular carcinomas may simply reflect their origin from adenomas, in which *ras* activation presumably plays a key early role, and may not be related directly to the differences in behaviour of the cancers.

It is not clear whether the subset of papillary cancers which *do* contain a mutant *ras* gene show any difference in biological behaviour, e.g. increased metastatic potential or a worse prognosis, compared to the majority. In our series there was no detectable correlation with any clinical or histopathological features but clearly a much larger, prospective study will be needed to establish this with certainty.

This study was supported by grants from the Cancer Research Campaign and the Welsh Office. We thank Dr Roseann Padua for synthesis of some of the oligonucleotides and for helpful discussions, and Kate Thomas for section cutting.

**References**

DEGROOT, L.J., LARSEN, P.R., REFETOFF, S. & STANBURY, J.B. (1984). *The Thyroid and Its Diseases*, p. 708. John Wiley: Chichester.

DENHARDT, D.T., GREENBURG, A.H., EGAN, S.E., HAMILTON, R.T. & WRIGHT, J.A. (1987). *Oncogene*, **2**, 55.

FRAZELL, F. & FOOTE, F.W. (1958). Papillary cancer of the thyroid–a review of 25 years experience. *Cancer*, **11**, 895.

FUSCO, A., GRIECO, M., SANTORO, M. & 5 others (1987). A new oncogene in human thyroid papillary carcinomas and their lymphnodal metastases. *Nature*, **328**, 170.

LEMOINE, N.R., MAYALL, E.S., WYLLIE, F.S. & 5 others (1988). Activated *ras* oncogenes in human thyroid cancers. *Cancer Res.*, **48**, 4459.

LEMOINE, N.R., MAYALL, E.S., WYLLIE, F.S. & 4 others (1989). High frequency of *ras* oncogene activation in all stages of human thyroid tumourigenesis. *Oncogene*, **4**, 159.

MUSCHEL, R.J., WILLIAMS, J.E., LOWY, D.R. & LIOTTA, L.A. (1985). Harvey *ras* induction of metastatic potential depends upon oncogene activation and the type of recipient cell. *Am. J. Pathol.*, **121**, 1.

SAIKI, R.K., BUGAWAN, T.L., HORN, G.T., MULLIS, K.B. & ERLICH, H.A. (1986). Analysis of enzymatically-amplified β-globin and HLA-DQα DNA with allele-specific oligonucleotide probes. *Nature*, **324**, 163.

SHIBATA, D.K., ARNHEIM, N. & MARTIN, W.J. (1988). Detection of human papilloma virus in paraffin-embedded tissue using the polymerase chain reaction. *J. Exp. Med.*, **167**, 225.

SUAREZ, H.G., DUVILLARD, J.A., CAILLOU, B. & 4 others (1988). Detection of activated *ras* oncogenes in human thyroid carcinomas. *Oncogene*, **2**, 403.

WOOD, W.I., GITSCHIER, J., LASKY, L.A. & LAWN, R.M. (1985). Base-composition-independent hybridisation in tetramethylammonium chloride: a method for oligonucleotide screening of highly complex gene libraries. *Proc. Natl Acad. Sci. USA*, **82**, 1585.